# Concomitant sarcoidosis, psoriasis, and eczema – immune patterns on the skin

**DOI:** 10.1186/s13023-024-03427-z

**Published:** 2024-10-30

**Authors:** Sebastian Sitaru, Alexander Zink, Tilo Biedermann, Susanne Annette Steimle-Grauer

**Affiliations:** School of Medicine and Health, Department of Dermatology and Allergy, Technical University of Munich, Munich, Germany

**Keywords:** Sarcoidosis, Eczema, Psoriasis, Autoimmunity, TNF alpha

## Abstract

Sarcoidosis is a rare and elusive chronic inflammatory disease. It can manifest itself in any organ, but preferentially affects the lungs and the skin. Our case of an elderly woman with cutaneous and pulmonary sarcoidosis presented with exacerbated itchy, scaly skin changes to our department. The clinical and histopathological findings were consistent with sarcoidosis and eczematized psoriasis. The case represents a unique presentation of sarcoidosis with cutaneous involvement and poses diagnostic and therapeutic challenges due to overlapping clinical features and histology covering the three diagnoses. We discuss the immunological complexities underlying this overlap and resulting possible treatment options, highlighting the role of dermatology in systemic autoimmune diseases involving the skin and the need for pathophysiology-based tailored management approaches in these diseases.

Sarcoidosis is a rare autoimmune disease of unknown etiology, which can affect all organs, but frequently manifests itself in the lungs and in the skin [1].

We present a case of a woman in her 70’s with a history of pulmonary sarcoidosis for 15 years and cutaneous involvement on the face and scalp for 5 years. The pulmonary involvement has been in radiological stage II since diagnosis and the patient did not complain of pulmonary or other systemic symptoms. Skin involvement on the scalp was treated with topical clobetasol 2–3 times a week. The pulmonary sarcoidosis was followed-up regularly by a specialist and did not require any other treatment. For two months before presenting to our department, the patient experienced progression of the skin changes, with an exacerbation of itch (numerical rating scale 8/10) and burning.

In our clinical examination, we found erythematous plaques with thick, psoriasiform scaling on the scalp, lower back, and retiform erythematous plaques with few scales on the forehead (Fig. 1A and B). Routine laboratory analysis results including creatinine, liver enzymes and complete blood count were unremarkable. Three punch biopsies for histopathological examination were obtained from the scalp and from the back. All biopsies showed multiple sarcoidal granulomas, periinfundibular spongiosis and lymphocytic infiltrate, as well as parakeratotic scaling in the epidermis, which is consistent with cutaneous sarcoidosis and co-existing seborrheic eczema with psoriasis-like scaling (Fig. 1C and D). The subjective symptoms and histology pointed to eczema, however the clinical appearance was typical of psoriasis, so we diagnosed a systemic sarcoidosis with cutaneous involvement, presenting as eczematized psoriasis. Because of failure of ultra-high potency topical steroids, we then planned a course of a tumor necrosis factor α (TNF-α) antagonist after obtaining insurance clearance.

Psoriasiform plaques as an extracutaneous manifestation of sarcoidosis have been described in case reports, but are overall considered extremely rare [2–4]. To our knowledge, our case is the first description of sarcoidosis with cutaneous involvement presenting as mixed psoriasis/eczema overlap. It not only highlights the unpredictable plethora of skin changes in sarcoidosis, but also the multifaceted role of the immune system in skin diseases.

Psoriasis and sarcoidosis can be triggered by Koebner phenomenon. Seborrheic eczema on the other hand can induce this phenomenon. This begs the question, which disease was first? Medical history suggests (systemic) sarcoidosis was first, and then skin changes in the form of eczematized psoriasis appeared, though this is subject to recall bias. From an immunological viewpoint however, there are common pathways for psoriasis and sarcoidosis, like the TNF-α axis, and for psoriasis and eczema, like interleukin 17-E (IL-17E) [5]. In psoriasis, IL-17 is produced by Th17 cells stimulated by IL-23, while TNF-α is involved at many points in its immune pathways, e.g. stimulating the release of IL-23 by dendritic cells [6]. In sarcoidosis, TNF-α produced by macrophages is thought to be involved in granuloma formation [7]. In addition to psoriasis, IL-17E also seems to be involved in eczema-type skin inflammation by mediating dendritic cell activation [8]. As we begin to deepen our understanding of the immune system pathways in inflammatory conditions, it becomes clear that biological systems often do not operate in binary modes, but that there are nuances and multiple pathways might be activated at the same time. Although in dermatology, different pathways can be observed as distinct patterns of skin changes, there are overlaps, and sometimes, as in our case, subjective symptoms, skin findings and histology cannot be attributed to one entity alone [5]. So perhaps the more relevant question is not which disease was first, but which pathways are activated and to which extent? Yet, cases similar to ours with macroscopic and microscopic features of multiple diseases can present a diagnostic, and by consequence, therapeutic challenge. Care should be taken to prioritize the diagnoses to avoid insufficiently treating the more severe diagnoses, in our case sarcoidosis. Because of the clinical appearance, and positive data for sarcoidosis and approval for psoriasis [9] in our case we opted for anti-TNF-α drugs. After three months of adalimumab 40 mg s.c. every two weeks, skin changes and subjective symptoms improved somewhat, but not resolved completely, suggesting the activation of the TNF-α pathway might not be the sole underlying immunological culprit.

In summary, our case reinforces that management of refractory skin changes should include multiple biopsies to gather information about the underlying immunology and plan treatment accordingly. Different disease entities should be prioritized according to their clinical relevance and severity to guide treatment decisions. The case also highlights the importance of dermatology for diagnosing systemic diseases and its potential to further unravel immunological phenomena through the careful assessment and analysis of skin changes and their underlying histopathology.

**Fig. 1:**
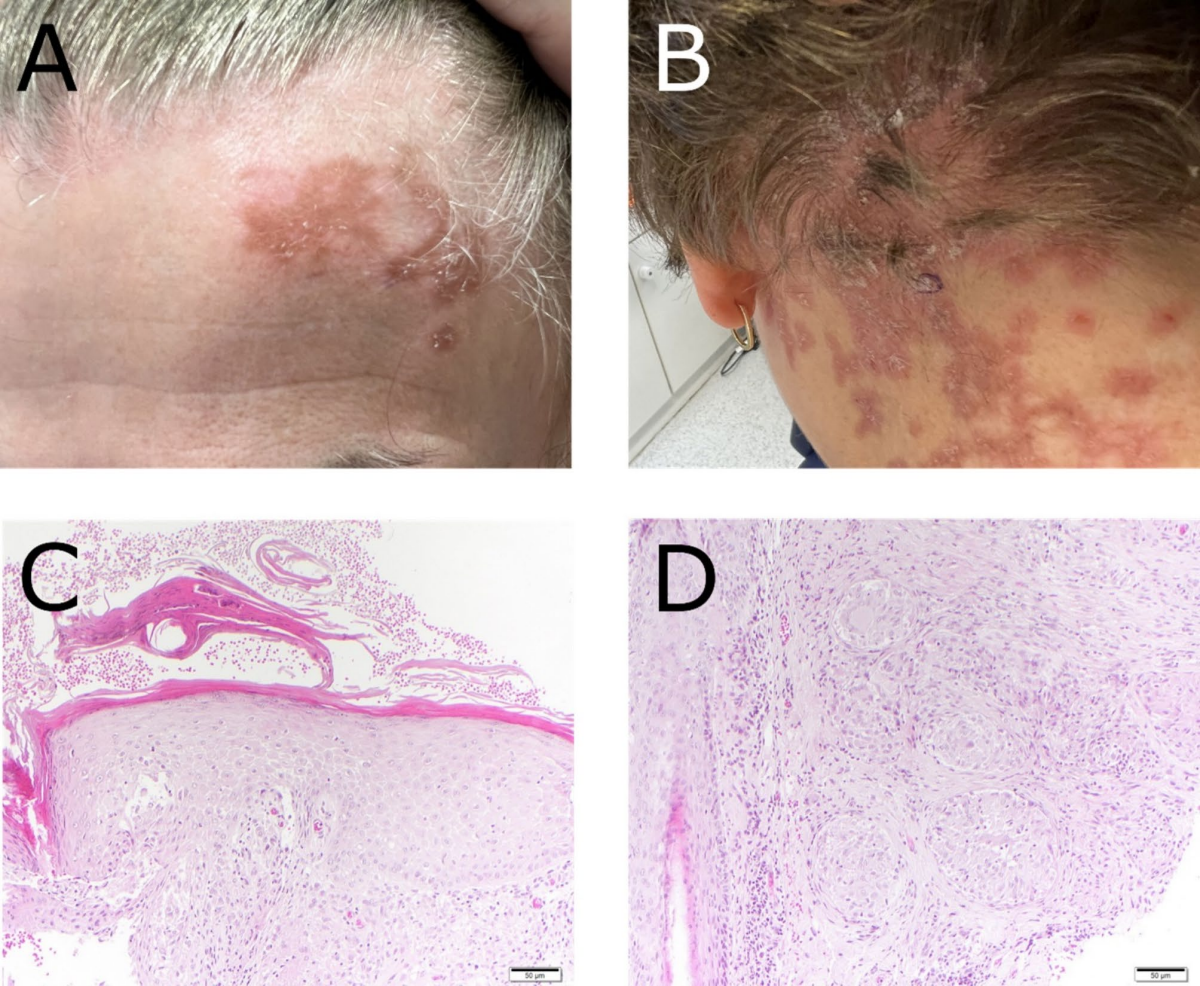
Skin and histopathological findings. **A** and **B**: erythematous plaques with silver scaling on the scalp/neck and the forehead. **C** and **D**: multiple sarcoidal granulomas of epitheloid and multinucleated giant cells with only few lymphocytes around them. Emphasis of the peri-infundibular region. Additionally, peri-infundibular spongiosis and a dense lymphocytic infiltrate. Both findings consistent with cutaneous sarcoidosis and co-existing seborrheic eczema with psoriasis-like scaling. **D**: Epidermis with parakeratotic scaling and a few included neutrophils

## Data Availability

All data supporting the findings of this study are available within the paper.

## Abbreviations

TNF-α: Tumor necrosis factor alpha
IL-23: Interleukin 23
IL-17: Interleukin 17

## References

[1] Haimovic A, Sanchez M, Judson MA, Prystowsky S, Sarcoidosis. A comprehensive review and update for the dermatologist: part I. cutaneous disease. J Am Acad Dermatol. 2012;66(5):699.e1-699.e18. 10.1016/j.jaad.2011.11.965 22507585

[2] Vega ML, Abrahams J, Keller M. Psoriasiform Sarcoidosis: collision of two entities or expression of one common pathogenesis? J Clin Aesthetic Dermatol. 2016;9(4):55–7. PMC4898586 27462388

[3] MITSUISHl T, Nogita T, Kawashima M. Psoriasiform Sarcoidosis with Ulceration. Int J Dermatol. 1992;31(5):339–40. 1587663 10.1111/j.1365-4362.1992.tb03950.x

[4] Argraves M, Sloan SB, Dadras SS. Cutaneous sarcoidosis masquerading as psoriatic plaques. Dermatol Online J. 2015;21(4):13030/qt0pq3r2qv. 25933072

[5] Eyerich K, Eyerich S. Immune response patterns in non-communicable inflammatory skin diseases. J Eur Acad Dermatol Venereol JEADV. 2018;32(5):692–703. 29114938 10.1111/jdv.14673 PMC5947562

[6] Guo J, Zhang H, Lin W, Lu L, Su J, Chen X. Signaling pathways and targeted therapies for psoriasis. Signal Transduct Target Ther. 2023;8(1):1–38. 38008779 10.1038/s41392-023-01655-6 PMC10679229

[7] Antoniu SA. Targeting the TNF-α pathway in sarcoidosis. Expert Opin Ther Targets. 2010;14(1):21–9. 20001207 10.1517/14728220903449244

[8] Lauffer F, Eyerich K. Eczematized psoriasis - a frequent but often neglected variant of plaque psoriasis. J Dtsch Dermatol Ges J Ger Soc Dermatol JDDG. 2023;21(5):445–53. 10.1111/ddg.14991 36772926

[9] Baeten D, van Hagen PM. Use of TNF blockers and other targeted therapies in rare refractory immune-mediated inflammatory diseases: evidence-based or rational? Ann Rheum Dis. 2010;69(12):2067–73. 20705637 10.1136/ard.2009.126813

